# Modeling Approaches to Predicting Persistent Hotspots in SCORE Studies for Gaining Control of Schistosomiasis Mansoni in Kenya and Tanzania

**DOI:** 10.1093/infdis/jiz529

**Published:** 2019-10-17

**Authors:** Ye Shen, Meng-Hsuan Sung, Charles H King, Sue Binder, Nupur Kittur, Christopher C Whalen, Daniel G Colley

**Affiliations:** 1 Department of Epidemiology and Biostatistics, College of Public Health, University of Georgia, Athens, Georgia, USA; 2 Center for Global Health and Diseases, Case Western Reserve University School of Medicine, Cleveland, Ohio, USA; 3 Schistosomiasis Consortium for Operational Research and Evaluation (SCORE), Center for Tropical and Emerging Global Diseases, University of Georgia, Athens, Georgia, USA; 4 Global Health Institute, University of Georgia, Athens, Georgia, USA; 5 Department of Microbiology, University of Georgia, Athens, Georgia, USA

**Keywords:** mass drug administration, modeling, persistent hotspots, praziquantel, schistosomiasis

## Abstract

**Background:**

Some villages, labeled “persistent hotspots (PHS),” fail to respond adequately in regard to prevalence and intensity of infection to mass drug administration (MDA) for schistosomiasis. Early identification of PHS, for example, before initiating or after 1 or 2 years of MDA could help guide programmatic decision making.

**Methods:**

In a study with multiple rounds of MDA, data collected before the third MDA were used to predict PHS. We assessed 6 predictive approaches using data from before MDA and after 2 rounds of annual MDA from Kenya and Tanzania.

**Results:**

Generalized linear models with variable selection possessed relatively stable performance compared with tree-based methods. Models applied to Kenya data alone or combined data from Kenya and Tanzania could reach over 80% predictive accuracy, whereas predicting PHS for Tanzania was challenging. Models developed from one country and validated in another failed to achieve satisfactory performance. Several Year-3 variables were identified as key predictors.

**Conclusions:**

Statistical models applied to Year-3 data could help predict PHS and guide program decisions, with infection intensity, prevalence of heavy infections (≥400 eggs/gram of feces), and total prevalence being particularly important factors. Additional studies including more variables and locations could help in developing generalizable models.

## Teaser

Mass drug administration for schistosomiasis identified persistent hotspots villages that failed to respond adequately in regard to prevalence and intensity of infection.We applied statistical models to predict such persistent hotspots and found several important factors.

Schistosomiasis, also known as bilharzia, is a parasitic disease caused by blood flukes of the genus *Schistosoma*. Schistosomiasis is most prevalent in tropical and subtropical areas and infects over 240 million people worldwide [1]. There are several strategies and treatments for controlling schistosomiasis, including preventive chemotherapy (PC) through mass drug administration (MDA) with praziquantel (PZQ), snail control with molluscicides, and access to safe water and sanitation [2]. Currently, the primary means of schistosomiasis control is PC through regular, periodic MDA with PZQ. The World Health Organization (WHO) recommends PC in endemic areas, mainly targeting school-aged children (SAC) [3].

The Schistosomiasis Consortium for Operational Research and Evaluation ([SCORE] https://score.uga.edu/) was established to conduct operational research to assist neglected tropical diseases program managers in controlling and preventing schistosomiasis. A major effort of SCORE has been large field studies to evaluate the effect of timing and alternative approaches to MDA on changes in prevalence and intensity of schistosomiasis. These studies took place in 5 sub-Saharan African countries [4].

In each of these studies, MDA resulted in significant decreases in average prevalence and intensity of infection by Year 5, when final parasitologic data were collected. However, a subset of villages in each study and in each study arm did not demonstrate expected decreases in prevalence and/or intensity of infection despite 2 or 4 years of MDA [5–7]. These nonresponding villages have been referred to as persistent hotspots (PHS) [6]. Being able to predict PHS is potentially useful to schistosomiasis control programs, because it might allow less intensive efforts in areas that are responding well to MDA and refocusing of resources on those that are not.

The objective of the analysis reported here is to evaluate 6 statistical approaches that might be used to try to predict the presence of PHS at Year 5 using data from Year 1, Year 3, or both Year 1 and Year 3 from large, 5-year SCORE field studies of gaining control of schistosomiasis mansoni in Kenya and Tanzania. The statistical approaches selected are among the most popular predictive models that are appropriate for our data. Through standardizing the coefficients of variables in the model and assessing their corresponding importance as inputs, we identified several crucial factors associated with the occurrence of PHS in the Kenya and Tanzania datasets.

## METHODS

### Study Design

Details of the SCORE multicenter studies of gaining and sustaining control of schistosomiasis have been previously published [8–10]. The analyses presented here use data from studies that took place in 150 villages in Kenya and 148 villages in Tanzania in areas around Lake Victoria having ≥25% prevalence of *Schistosoma mansoni* infection among SAC.

**Figure 1.**
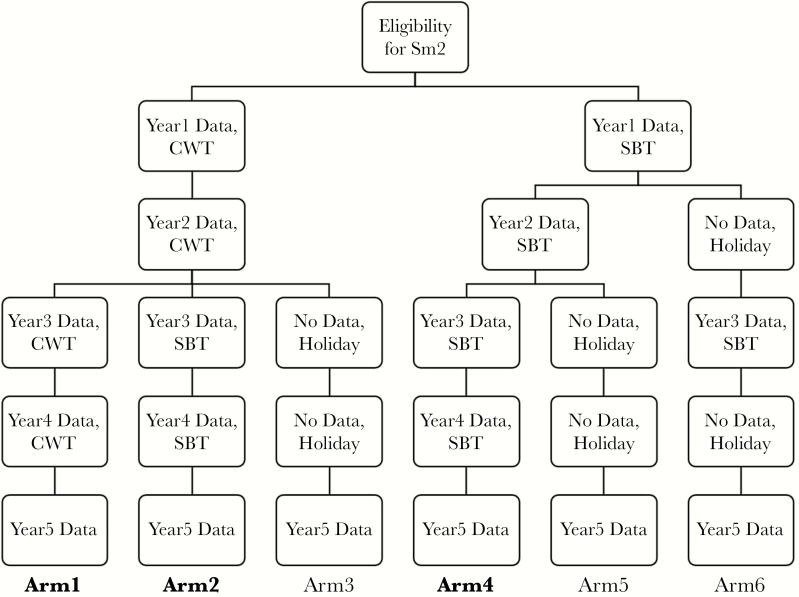
Study arms and timeline for the studies of gaining control of schistosomiasis from SCORE.

In both the Kenya and Tanzania studies, villages were randomized to 1 of 6 arms (Figure 1). Mass drug administration in these studies either involved school-based treatment (SBT) or community-wide treatment (CWT). In SBT, the protocol called for teachers to deliver PZQ to SAC and directly observe the students swallowing the treatment. In CWT villages, treatment was provided to all eligible persons in the village. In Years 1 and 2, CWT was conducted by community drug distributors, who distributed PZQ by going house-to-house. In Years 3 and 4, in addition to house-to-house distribution, in CWT villages PZQ was delivered in schools by teachers.

Parasitologic surveys were conducted annually, either shortly before or during MDA campaigns. The analyses in the current study use only the data from Arms 1, 2, and 4—those arms that received annual MDA with PZQ and had annual parasitologic surveys. The main study outcomes were prevalence and intensity of *S mansoni* infection among 9- to 12-year-old children. Village-level treatment coverage was measured during or shortly after each MDA and was defined as the percentage of SAC in the village who had received MDA.

### Persistent Hotspots

For our analyses, we defined a PHS as a village that failed to reduce its *S mansoni* prevalence among 9- to 12-year-old children by at least 35% and/or failed to reduce its mean intensity of infection among SAC by at least 50% from baseline to Year 5. Maps of PHS in Kenya and Tanzania have been previously published in Figure 4 of Kittur et al [11].

### Parasitological Data

During each parasitologic survey, up to 3 stool specimens were collected from 100 9- to 12-year-old children per village. *Schistosoma mansoni* infection was diagnosed by microscopic examination of stool specimens using duplicate Kato-Katz thick smears. The prevalence of infection was defined as the proportion of participants in each village who were egg-positive. A study participant’s infection intensity was determined by averaging the numbers of eggs observed in each of their Kato-Katz slides (up to 6 slides per child). Actual egg counts were converted to eggs per gram (epg) by multiplying counts by 24 [12]. We used the actual epg in most analyses, as well as evaluating whether prevalence of infection intensity >200 epg and prevalence of heavy infection (defined by WHO as ≥400 epg) [13] could serve as useful predictors.

### Statistical Analysis

We applied 6 different predictive approaches covering 2 major categories: (1) the more conventional regression approaches including the traditional logistic regression (lgt) [14, 15] and its 2 extensions with variable selection procedures in elastic-net logistic regression (logit) [16], and logistic regression with LASSO (LASSO) [17], and (2) the tree-based machine learning methods including gradient boosting machine (GBM) [18, 19], random forests (rf) [20], and single decision tree (tree) [21]. We then assessed their corresponding performances in terms of the accuracy of predicting PHS/non-PHS. Accuracy was defined as the percentage of Year 5 PHS and/or non-PHS that were predicted correctly, calculated as (1-missclassification error). R software version 3.5.1 [22] was used for statistical computing.

Five potential predictors were considered in the full models, including prevalence, intensity, coverage, and prevalence of infections with epg ≥ 200 (gt200) and with epg ≥ 400 (gt400); each of these were evaluated using only Year 1 or Year 3 data and also using both years’ data together in models. Results are reported as (1) the mean value of prediction accuracies through replicated splits of training and validation datasets or (2) a single prediction accuracy if both training and validation datasets were given and fixed. Most models included diagnostics to detect variables that profoundly influenced the prediction procedure, except for the traditional logistic and single decision tree approaches, where the prediction performances were consistently outperformed by other methods. The variable importance in GBM and rf was determined by calculating the relative influence of each variable measured by the percentage of variance contribution [23]. Decisions about which features to split were based on which reduced a node’s squared error the most—specifically, how much the squared error over all trees is reduced as GBM/rf splits on that variable. Variable importance for the elastic-net logistic regression and the logistic regression model with LASSO was assessed by the magnitude of the standard coefficient for comparison purposes [16].

Three different scenarios were designed to evaluate the performances of the 6 statistical approaches (Table 1). In Scenario 1, 70% of the observations from a given country (Kenya or Tanzania) were randomly sampled to create a training dataset, and the other 30% of observations from the same country were used as a validation dataset. A random sample split with the same cross-validation mechanism was repeated 200 times to obtain a reliable mean value of accuracy. In Scenario 2, to explore the external validities of the predictive models across countries, we trained the models with 100% of the data from one country and validated it with 100% of the data from the other.

**Table 1.** Study Scenarios for Examining Accuracy of Model Prediction in SCORE Studies of Gaining Control of *Schistosoma mansoni* in Kenya and Tanzania

Dataset	Training	Validation
Scenario 1	70% of a single country’s dataset	30% of the same country’s dataset
Scenario 2	100% of one country’s dataset	100% of the other country’s dataset
Scenario 3	70% of the combined Kenya and Tanzania dataset	30% of the combined Kenya and Tanzania dataset

We also conducted a sensitivity analysis to see whether the proportion of PHS in the training dataset affects model performance. We labeled all villages in the Tanzania and Kenya datasets as PHS or non-PHS according to our definition. To create a “high-PHS training dataset” for each country, we randomly picked 70% of PHS villages and 30% of non-PHS villages and used the rest of the villages as validation datasets. For “low-PHS training datasets”, we randomly picked 30% of PHS villages and 70% of non-PHS villages and left the rest of the villages in the validation dataset.

In Scenarios 3, we used 70% of the data from a pooled Kenya-Tanzania dataset for training and 30% for validation, first without and then with a dummy variable included to provide a country label in the models. These scenarios were first implemented using data from Year 1 from Kenya and Tanzania for prediction, then using combined Year 1 and Year 3 data, and then using Year 3 data alone.

### Ethics Statement

The original study protocol that provided the data analyzed in this study was approved by institutional review panels in each African country and by their academic partners. The University of Georgia (UGA) Institutional Review Board conducted an administrative review of each study to ensure that all individual panel reviews met UGA human subjects protection requirements. Parents of children participating in the study provided written informed consent, and assent was obtained from child participants.

## RESULTS

### Predictive Performance of Models

We obtained the descriptive statistics for the villages studied (Table 2). Over the 5 years of the study, prevalence, intensity, and prevalence of high-intensity infections decreased in both countries, with a larger decline in Kenya. Persistent hotspot prevalence at Year 5 was markedly different in the 2 countries: 70% in Tanzania and 25% in Kenya.

**Table 2.** Means and 95% CIs for Key Predictors and PHS Percentages in SCORE Studies of Gaining Control of *Shistosoma mansoni* in Kenya and Tanzania

Country	Measure	Year 1	Year 3	Year 5
Kenya (75 villages)	Prevalence	60% (55%–66%)	40% (34%–46%)	26% (20%–32%)
	gt200^a^	14% (10%–18%)	9% (5%–12%)	4% (2%–6%)
	gt400^b^	7% (4%–9%)	4% (2%–6%)	2% (1%–3%)
	Intensity (eggs per gram)	87 (65–110)	54 (36–72)	30 (16–45)
	Coverage^c^	84% (67%–101%)	90% (88%–92%)	
	PHS prevalence			**25% (19/75** **)**
Tanzania (74 villages)	Prevalence	56% (49%–63%)	38% (32%–44%)	43% (36%–50%)
	gt200^a^	17% (13%–21%)	6% (4%–8%)	6% (4%–9%)
	gt400^b^	9% (6%–12%)	2% (1%–3%)	3% (2%–4%)
	Intensity (eggs per gram)	124 (92–156)	41 (31–51)	46 (33–59)
	Coverage^c^	79% (76%–81%)	75% (73%–77%)	
	PHS prevalence			**70% (52/74)**

Percentage of PHS villages in bold.

Abbreviations: CI, confidence interval; epg, eggs per gram; MDA, mass drug administration; PHS, persistent hotspots; SCORE, Schistosomiasis Consortium for Operational Research and Evaluation.

^a^gt200: prevalence of intensity that was greater than 200 epg

^b^gt400: prevalence of intensity that was greater than 400 epg.

^c^Coverage: the percentage of school-aged children who received MDA. Note that there was no treatment in Year 5, and hence no coverage data for Year 5.

In general, generalized linear models (GLMs) with variable selection procedures, including elastic-net and LASSO, possessed relatively stable performance compared with the tree-based machine learning (tree/rf/GBM) methods. In terms of prediction with Kenya Year 1 data in Scenario 1, most methods possessed ~80% prediction accuracy for PHS at Year 5, except the single-tree method, which had an accuracy of only ~65%. By contrast, with Tanzania Year 1 data in Scenario 1, all modeling methods performed similarly, at slightly over 70% prediction accuracy (Supplementary Table S1). Other scenarios also suggest that using only Year 1 data did not lead to satisfactory prediction performances.

Predictive performances using both Year 1 and Year 3 data are evaluated (Table 3). In Scenario 1, which uses a single country’s data for both training and validation, the addition of data from Year 3 improved most model performances to over 85% accuracy in Kenya, but it only slightly increased the prediction accuracy with Tanzania data. The traditional logistic regression was not the worst strategy in our examination; in fact, this conventional method seemed to outperform the single-tree method. Generalized linear models generally performed well (85% and above prediction accuracies) when validating with the Kenya dataset, but they only achieved ~70% accuracies when the validation dataset was from Tanzania.

**Table 3.** Mean Prediction Accuracy Among 6 Models, by Scenario, Using Year 1 and Year 3 Data

Comparison	GBM	rf	tree	logit	LASSO	lgt
Scenario 1						
Kenya data only	0.90	0.91	0.79	0.90	0.90	0.86
Tanzania data only	0.75	0.76	0.71	0.72	0.72	0.72
Scenario 2						
Training with Kenya, predicting Tanzania	0.70	0.70	0.70	0.73	0.72	0.72
Training with Tanzania, predicting Kenya	0.51	0.59	0.48	0.89	0.89	0.81
Scenario 3						
Without country label	0.76	0.77	0.61	0.73	0.73	0.72
With country label	0.79	0.80	0.65	0.80	0.81	0.78

Abbreviations: GBM, gradient boosting machine; LASSO, logistic regression with LASSO; lgt, traditional logistic regression; logit, elastic-net logistic regression; rf, random forests; tree, single decision tree

Under Scenario 2, models trained from one country’s Year 1 and Year 3 dataset to predict another country’s PHS villages were less effective. This was especially true with the tree-based machine learning approaches (tree/rf/GBM) (Table 3).

Given that Tanzania had 70% PHS, compared with 25% in Kenya, we investigated whether differences in PHS prevalence could have contributed to poor performance in Scenario 2. As an experiment, we conducted separate analyses for Kenya and Tanzania in which we split each country’s datasets to create “high-PHS” and “low-PHS” training datasets as described in the methods. Supplementary Table S2 shows that modifying the training and validation datasets this way does not lead to similar results as shown in Scenario 2, suggesting that factors besides the percentage of PHS may be important in developing models using data from one area or country to develop models for another.

In Scenario 3, we trained and validated with the combined Years 1 and 3 Kenya and Tanzania dataset. Prediction results were similar to that of the Scenario 1 Tanzania-only assessment, except for the tree-based method, which performed worse. By adding a variable to adjust for the country factor, we improved prediction in all models. This suggests that the country label could be important when data from multiple countries or geographic areas are combined for training and prediction.

We also trained with the combined Kenya and Tanzania datasets and then validated first with the Kenya and then with the Tanzania datasets. As expected, all the machine learning-based approaches (tree/rf/GBM) achieved “perfect” accuracy under this situation (validating with a country’s own subset). On the other hand, even though GLMs performed well (over 90%) for validating with the unbalanced Kenya dataset, validation with the Tanzania dataset had only 70% accuracy using validation with the Tanzania dataset (Supplementary Table S3). In a separate analysis, using only Year 3 data for PHS prediction yields a similar pattern (Table 4) to that observed using Year 1 and Year 3 data (Table 3). Some models even outperformed those trained from both Year 1 and Year 3 data, which suggests that the Year 3 data could be influential in predicting the PHS villages at Year 5.

**Table 4.** Mean Prediction Accuracy Among 6 Models, by Scenario, Using Year 3 Data Alone

Comparison	GBM	rf	tree	logit	LASSO	lgt
Scenario 1						
Kenya data only	0.89	0.89	0.77	0.91	0.91	0.89
Tanzania data only	0.74	0.75	0.72	0.74	0.74	0.72
Scenario 2						
Training with Kenya, predicting Tanzania	0.72	0.70	0.70	0.72	0.74	0.70
Training with Tanzania, predicting Kenya	0.53	0.64	0.35	0.92	0.92	0.92
Scenario 3						
Without country label	0.73	0.72	0.59	0.71	0.71	0.71
With country label	0.79	0.78	0.65	0.81	0.82	0.81

Abbreviations: GBM, gradient boosting machine; LASSO, logistic regression with LASSO; lgt, traditional logistic regression; logit, elastic-net logistic regression; rf, random forests; tree, single decision tree.

### Variable Importance

To better understand the pivotal factors in predicting PHS, the variables used in our models were further evaluated for their relative utility for PHS prediction. In this substudy, we focused on the 4 predictive approaches that performed best in the preceding analyses: GBM, rf, GLMs with elastic-net, and LASSO. We checked the rank of variable importance in models using datasets that included Year 1 and Year 3 data, then models including only Year 3 data. Important variables as measured by their corresponding variance contributions across various models and datasets are listed in Table 5.

**Table 5.** Variables Demonstrated to Be Important Across Various Models and Datasets

Datasets	Years from which predictors were used	Models		
		GBM	Random Forests	GLMs (Elastic-net and LASSO)
Kenya	Y1 and Y3	• Y3 Intensity	• Y3 Intensity	• Y3 Intensity
		• Y3 gt400	• Y3 gt400	• Y3 gt400
	Y3	• Y3 gt400	• Y3 Intensity	• Y3 gt200
			• Y3 gt400	• Y3 gt400
Tanzania	Y1 and Y3	• Y3 Intensity	• Y3 Intensity	• Y3 Intensity
		• Y1 Coverage	• Y1 Coverage	• Y1 Coverage
		• Y1 gt400	• Y1 gt400	• Y3 Prevalence
	Y3	• Y3 Intensity	• Y3 Intensity	• Y3 Prevalence
		• Y3 Prevalence	• Y3 Prevalence	• Y3 gt400
Kenya and Tanzania (without country label)	Y1 and Y3	• Y3 Intensity	• Y3 Coverage	• Y3 Intensity
		• Y3 Coverage	• Y1 Intensity	• Y1 Intensity
		• Y1 Intensity	• Y3 gt400	• Y3 gt400
	Y3	• Y3 Coverage	• Y3 Coverage	• Y3 Intensity
		• Y3 Intensity	• Y3 Intensity	• Y3 gt400
		• Y3 gt400	• Y3 gt400	
Kenya and Tanzania (with country label)	Y1 and Y3	• Y3 Intensity	• Y3 Intensity	• Y3 Intensity
		• Country	• Country	• Country
				• Y3 gt400
	Y3	• Y3 Intensity	• Y3 Intensity	• Y3 Intensity
		• Country	• Country	• Country
			• Y3 gt200	• Y3 gt400

Abbreviations: GBM, gradient boosting machine; GLM, generalized linear model; LASSO, logistic regression with LASSO; Y, year.

Using Kenya’s Year 1 and Year 3 data, Year 3 infection intensity and Year 3 gt400 (prevalence of heavy infections) were the prominent variables in both GBM and rf approaches. These 2 variables had close to 80% overall influence in the GBM’s splitting decision and close to 50% overall influence in the rf. They were also the common variables in the GLMs with elastic-net and LASSO. The standardized coefficients of Year 3 intensity and Year 3 gt400 exhibited strong positive associations with PHS status (Supplementary Tables S4 and S5). With Kenya’s Year 3 data only, Year 3 gt400 in Year 3 dominated, with over 80% overall influence in the GBM. Year 3 infection intensity and Year 3 gt400 each contribute 31% overall influence (a combined total of 62%) in the rf. Meanwhile, Year 3 gt200 and Year 3 gt400 were important predictors in both GLMs with elastic-net and LASSO, but the Year 3 intensity was also positively associated with PHS status in the elastic-net logistic regression (Supplementary Tables S6 and S7).

 For the Tanzania dataset including Year 1 and Year 3 data, Year 3 intensity, Year 1 coverage, and Year 1 gt400 were the major influences in both GBM and rf approaches. The 3 variables combined had close to 60% overall influence in the GBM’s splitting decision and in the rf. Meanwhile, Year 1 coverage and Year 3 prevalence were the common variables selected from the elastic-net logistic regression and logistic regression with LASSO to be positively associated with PHS status (Supplementary Tables S8 and S9). With Tanzania’s Year 3 data only, intensity and prevalence were the vital variables in both GBM and rf approaches; 75% and 60% of combined influence in the GBM and rf approaches were attributed to these 2 factors, respectively. Besides Year 3 prevalence, Year 3 gt400 was also selected as an important predictor from both elastic-net logistic regression and logistic regression with LASSO (Supplementary Tables S10 and S11).

 In addition, we studied whether the identified important variables would be different among models with the combined data from Kenya and Tanzania. When adding a country label into the models using Years 1 and 3 data pooled together, the results of GBM showed that Year 3 intensity and the country factor possessed over 50% of overall influence. In rf, 2 additional variables—Year 3 coverage and Year 1 intensity—were needed to achieve over 50% total importance. For GLMs, the country factor, Year 3 intensity, and Year 3 gt400 were strongly associated with PHS status (Supplementary Tables S12 and S13). In contrast, when we excluded the country factor from the models, Year 3 coverage, Year 3 intensity, Year 1 intensity, and Year 3 gt400 were among the most important factors in GBM and rf. Similar influential predictors were identified in models that did not contain the country factor, with Year 1 intensity increasing in influence (Supplementary Tables S16 and S17).

When using only the Year 3 data, the country factor seemed not as influential. Gradient boosting machine with the country label showed that Year 3 intensity contributed almost 50% of overall influence among all considered variables. In the rf, Year 3 intensity and Year 3 gt200 controlled half of the importance within the model. Furthermore, Year 3 intensity, country factor, and Year 3 gt400 were selected as important predictors from the logistic regressions with elastic-net and with LASSO (Supplementary Tables S14 and S15). When the country label was excluded, Year 3 intensity and Year 3 gt400 had the most influence among GBM, rf, and GLMs (Supplementary Tables S18 and S19).

## DISCUSSION

We applied 3 different approaches to predicting PHS using Year 1 and 3 data from Kenya and Tanzania collected through the 5-year SCORE studies for gaining control of schistosomiasis mansoni. Overall, GBM, rf, elastic-net logistic regression, and logistic regression with LASSO outperformed single decision tree and traditional logistic regression. Generalized linear models with variable selections possessed relatively stable performances in predicting PHS, regardless of how we designed training and validation datasets. Under each of the scenarios, GLMs methods had prediction accuracy over 70%. The accuracy of tree-based approaches can decline sharply if the training and validation datasets are not from the same country.

For all of the approaches, models using Year 1 (baseline) data alone had some success in predicting PHS. Year 2 data alone, or adding Year 2 data to the Year 1 and Year 3 dataset, did not improve prediction (data not shown). Addition of data collected after 2 rounds of MDA (Year 3 data) greatly improved predictions; however, use of Year 3 data alone may be adequate for prediction in many settings.

The variable importance assessment suggested that infection intensity at Year 3 was a key factor, because it was highly associated with Year 5 village PHS status. In addition, prevalence of heavy infections (≥400 epg) at Year 3 and total prevalence in Year 3 were frequently influential predictors. Although these variables are not completely independent, for purposes of prediction it is appropriate to consider them as distinct inputs. Future field studies should evaluate the utility of these factors for predicting PHS, perhaps as early as in Year 3, after 2 rounds of annual MDA [24, 25].

When the training and validating sources were from different countries (Scenario 2), the advantages of GLMs were clear. However, as shown in Supplementary Table S2, differences in PHS prevalence alone did not explain the observed discrepancies in prediction accuracies. With split training and validation datasets at different levels of PHS prevalence, the GLMs had comparable performances to the tree-based approaches in Kenya but were outperformed by tree-based models in Tanzania, suggesting heterogeneities at the country level. It is also likely that the GLMs were able to retain stable performances by tuning the F1 optimal thresholds, which could have helped to avoid misclassifications, whereas the tree-based methods seemed to lack a mechanism to handle heterogeneities between the training and validation datasets. As a result, the tree/rf/GBM methods obtained relatively poor performances in Scenario 2.

 The Kenya and Tanzania studies were carried out in discrete but sizable areas around Lake Victoria. Our work demonstrates that it may be possible to develop good models for predicting PHS in a large area in a single country. However, using data from one country to predict PHS in another may be more challenging. Models including the country factor (whether in the tree-based methods or GLMs) revealed that close to 20% of total influence is attributable to the difference in study settings, suggesting a significant level of heterogeneity between these settings in these countries.

Our study is limited by the relatively few variables with which we could explore such heterogeneity and by being limited to datasets from only 1 area in each of 2 countries. It would be useful to have similar data from additional countries or areas of Kenya and Tanzania, to assess whether countries “lump” into categories that allow for better multisite prediction. Future studies that include data on village-level behavioral or environmental risk factors and data from additional areas may lead to more generalizable models.

An interesting finding from our analyses was that models using full or partial Tanzania data for validation almost always underperformed those using Kenya data. Even when the training and validation datasets were both from Tanzania, prediction accuracy was significantly lower than in models validated by Kenya data. In Year 5 of the SCORE study, many villages in Tanzania experienced an increase in prevalence compared with Year 4, reversing the pattern seen in all previous years [26]. An anomalous year or other unmeasured factors, such as the distance between villages and closest water resources or population shifts, were not considered in the descriptive models described in this paper. 

## CONCLUSIONS

Our analysis of multiple models demonstrates the feasibility of developing prediction algorithms for PHS that could potentially help program managers adjust multiyear MDAs after as little as 2 years of implementation, to maximize impact.

## Supplementary Data

Supplementary materials are available at The *Journal of Infectious Diseases* online. Consisting of data provided by the authors to benefit the reader, the posted materials are not copyedited and are the sole responsibility of the authors, so questions or comments should be addressed to the corresponding author.

**Table S1.** Mean prediction accuracy among 6 models, by Scenario, using Year 1 data.

**Table S2.** Examination of mean accuracy, using different proportions of PHS in training and validation datasets with Kenya and Tanzania data.

**Table S3.** Examination of mean accuracy, using multicountry data as training and one of countries as validation.

**Table S4.** Variable importance of GBM and rf with Kenya Year 1 and Year 3 data.

**Table S5.** Standardized coefficients of GLMs with Kenya Year 1 and Year 3 data.

**Table S6.** Variable importance of GBM and rf with Kenya Year 3 data.

**Table S7.** Standardized coefficients of GLMs with Kenya Year 3 data.

**Table S8.** Variable importance of GBM and rf with Tanzania Year 1 and Year 3 data.

**Table S9.** Standardized coefficients of GLMs with Tanzania Year 1 and Year 3 data.

**Table S10.** Variable importance of GBM and rf with Tanzania Year 3 data.

**Table S12.** Variable importance of GBM and rf with Kenya and Tanzania Year 1 and Year 3 data (with Country label).

**Table S13.** Standardized coefficients of GLMs with Kenya and Tanzania Year 1 and Year 3 data (with Country label).

**Table S14.** Variable importance of GBM and rf with Kenya and Tanzania Year 3 data (with Country label).

**Table S15.** Standardized coefficients of GLMs with Kenya and Tanzania Year 3 data (with Country label).

**Table S16.** Variable importance of GBM and rf with Kenya and Tanzania Year 1 and Year 3 data.

**Table S17.** Standardized coefficients of GLMs with Kenya and Tanzania Year 1 and Year 3 data.

**Table S18.** Variable importance of GBM and rf with Kenya and Tanzania Year 3 data.

**Table S19.** Standardized coefficients of GLMs with Kenya and Tanzania Year 3 data.

jiz529_suppl_Supplementary_Tables

## Notes

***Disclaimer.*** The funders had no role in the study design, data collection and analysis, decision to publish, or preparation of the manuscript. The authors declare that the research was conducted in the absence of any commercial or financial relationships that could be construed as a potential conflict of interest.

***Financial support.*** This study received financial support from University of Georgia Research Foundation, Inc., which was funded by the Bill & Melinda Gates Foundation for the Schistosomiasis Consortium for Operational Research and Evaluation (SCORE) project.

***Potential conflicts of interest.*** All authors: No reported conflicts of interest. All authors have submitted the ICMJE Form for Disclosure of Potential Conflicts of Interest.
