# Supplementary material for: Modeling Approaches to Predicting Persistent Hotspots in SCORE Studies for Gaining Control of Schistosomiasis Mansoni in Kenya and Tanzania
Source: J Infect Dis. 2019 Oct 17;221(5):796–803. doi: 10.1093/infdis/jiz529 (PMC7026890; doi:10.1093/infdis/jiz529)
Supplement: jiz529_suppl_Supplementary_Tables [file jiz529_suppl_supplementary_tables.doc]

**Supplementary Tables**

Table S1. Mean prediction accuracy among six models, by Scenario, using Year 1 data

| Comparison | GBM | rf | tree | logit | LASSO | lgt |
| --- | --- | --- | --- | --- | --- | --- |
| **Scenario 1** |  |  |  |  |  |  |
| Kenya data only | 0.79 | 0.83 | 0.65 | 0.83 | 0.83 | 0.80 |
| Tanzania data only | 0.74 | 0.74 | 0.71 | 0.71 | 0.71 | 0.71 |
| **Scenario 2** |  |  |  |  |  |  |
| Training with Kenya, predicting Tanzania | 0.70 | 0.72 | 0.70 | 0.70 | 0.70 | 0.70 |
| Training with Tanzania, predicting Kenya | 0.51 | 0.25 | 0.25 | 0.27 | 0.27 | 0.25 |
| **Scenario 3** |  |  |  |  |  |  |
| Without country label | 0.63 | 0.62 | 0.52 | 0.60 | 0.61 | 0.61 |
| With country label | 0.74 | 0.78 | 0.60 | 0.78 | 0.78 | 0.78 |

Table S2. Examination of mean accuracy, using different proportions of PHS in training and validation datasets with Kenya and Tanzania data

| Comparison | GBM | rf | tree | logit | LASSO | lgt |
| --- | --- | --- | --- | --- | --- | --- |
| Kenya data only, high-PHS training dataset | 0.89 | 0.96 | 0.71 | 0.96 | 0.96 | 0.93 |
| Kenya data only, low PHS training dataset | 0.81 | 0.55 | 0.48 | 0.48 | 0.48 | 0.48 |
| Tanzania data only, high-PHS training dataset | 0.77 | 0.87 | 0.77 | 0.87 | 0.83 | 0.80 |
| Tanzania data only, low-PHS training dataset | 0.86 | 0.86 | 0.86 | 0.86 | 0.86 | 0.40 |

Table S3. Examination of mean accuracy, using multi-country data as training and one of countries as validation

| Comparison | GBM | rf | tree | logit | LASSO | lgt |
| --- | --- | --- | --- | --- | --- | --- |
| Training with the combined dataset, validating with Kenya | 1 | 1 | 1 | 0.91 | 0.92 | 0.92 |
| Training with the combined dataset, validating with Tanzania | 1 | 1 | 1 | 0.70 | 0.70 | 0.72 |

Table S4. Variable importance of GBM and rf with Kenya Year 1 and Year 3 data

| Variable | Percentage of variance contribution  (GBM) | Percentage of variance contribution (rf) |
| --- | --- | --- |
| Y1 Prevalence | 0.008 | 0.06 |
| Y1 gt200 | 0.04 | 0.07 |
| Y1 gt400 | 0.01 | 0.02 |
| Y1 Intensity | 0.07 | 0.05 |
| Y1 Coverage | 0.06 | 0.03 |
| Y3 Prevalence | 0 | 0.07 |
| Y3 gt200 | 0.003 | 0.17 |
| Y3 gt400 | 0.23 | 0.33 |
| Y3 Intensity | 0.57 | 0.17 |
| Y3 Coverage | 0.007 | 0.05 |

*Y1/Y3 Prevalence: Prevalence of Year 1 or Year 3

*Y1/Y3 Intensity: Intensity of Year 1 or Year 3

*Y1/Y3 gt200: Prevalence of intensity that was greater than 200 epg in Year 1 or Year 3

*Y1/Y3 gt400: Prevalence of intensity that was greater than 400 epg in Year 1 or Year 3

*Y1/Y3 Coverage: The percentage of School-aged children who received MDA in Year 1 or Year 3

Table S5. Standardized coefficients of GLMs with Kenya year 1 and year 3 data

| Variable | Coefficients  (Elastic-net logistic) | Coefficients  (LASSO) |
| --- | --- | --- |
| Y1 Prevalence | -0.35 | -0.75 |
| Y1 gt200 | 0 | 0 |
| Y1 gt400 | 0 | 0 |
| Y1 Intensity | 0 | 0 |
| Y1 Coverage | 0 | 0 |
| Y3 Prevalence | 0.11 | 0 |
| Y3 gt200 | 0.51 | 0 |
| Y3 gt400 | 0.59 | 0.48 |
| Y3 Intensity | 0.64 | 2.04 |
| Y3 Coverage | 0 | 0.02 |

*Y1/Y3 Prevalence: Prevalence of year 1 or year 3

*Y1/Y3 Intensity: Intensity of year 1 or year 3

*Y1/Y3 gt200: Prevalence of intensity that was greater than 200 epg in year 1 or year 3

*Y1/Y3 gt400: Prevalence of intensity that was greater than 400 epg in year 1 or year 3

*Y1/Y3 Coverage: The percentage of School-aged children who received MDA in year 1 or year 3

Table S6. Variable importance of GBM and rf with Kenya year 3 data

| Variable | Percentage of variance contribution  (GBM) | Percentage of variance contribution (rf) |
| --- | --- | --- |
| Y3 Prevalence | 0.09 | 0.13 |
| Y3 gt200 | 0 | 0.17 |
| Y3 gt400 | 0.85 | 0.31 |
| Y3 Intensity | 0.002 | 0.31 |
| Y3 Coverage | 0.06 | 0.07 |

*Y3 Prevalence: Prevalence of year 3

*Y3 Intensity: Intensity of year 3

*Y3 gt200: Prevalence of intensity that was greater than 200 epg in year 3

*Y3 gt400: Prevalence of intensity that was greater than 400 epg in year 3

*Y3 Coverage: The percentage of School-aged children who received MDA in year 3

Table S7. Standardized coefficients of GLMs with Kenya year 3 data

| Variable | Coefficients  (Elastic-net logistic) | Coefficients  (LASSO) |
| --- | --- | --- |
| Y3 Prevalence | 0.03 | 0 |
| Y3 gt200 | 0.59 | 0.14 |
| Y3 gt400 | 0.70 | 2.41 |
| Y3 Intensity | 0.53 | 0 |
| Y3 Coverage | -0.05 | 0 |

*Y3 Prevalence: Prevalence of year 3

*Y3 Intensity: Intensity of year 3

*Y3 gt200: Prevalence of intensity that was greater than 200 epg in year 3

*Y3 gt400: Prevalence of intensity that was greater than 400 epg in year 3

*Y3 Coverage: The percentage of School-aged children who received MDA in year 3

Table S8. Variable importance of GBM and rf with Tanzania Year 1 and Year 3 data

| Variable | Percentage of variance contribution  (GBM) | Percentage of variance contribution (rf) |
| --- | --- | --- |
| Y1 Prevalence | 0.04 | 0.06 |
| Y1 gt200 | 0.05 | 0.11 |
| Y1 gt400 | 0.17 | 0.12 |
| Y1 Intensity | 0.04 | 0.12 |
| Y1 Coverage | 0.20 | 0.13 |
| Y3 Prevalence | 0.09 | 0.09 |
| Y3 gt200 | 0 | 0.05 |
| Y3 gt400 | 0 | 0.02 |
| Y3 Intensity | 0.25 | 0.22 |
| Y3 Coverage | 0.15 | 0.08 |

*Y1/Y3 Prevalence: Prevalence of Year 1 or Year 3

*Y1/Y3 Intensity: Intensity of Year 1 or Year 3

*Y1/Y3 gt200: Prevalence of intensity that was greater than 200 epg in Year 1 or Year 3

*Y1/Y3 gt400: Prevalence of intensity that was greater than 400 epg in Year 1 or Year 3

*Y1/Y3 Coverage: The percentage of School-aged children who received MDA in Year 1 or Year 3

Table S9. Standardized coefficients of GLMs with Tanzania year 1 and year 3 data

| Variable | Coefficients  (Elastic-net logistic) | Coefficients  (LASSO) |
| --- | --- | --- |
| Y1 Prevalence | -0.38 | -0.46 |
| Y1 gt200 | 0 | 0 |
| Y1 gt400 | -0.33 | -0.43 |
| Y1 Intensity | 0 | 0.14 |
| Y1 Coverage | 0.25 | 0.27 |
| Y3 Prevalence | 0.46 | 0.54 |
| Y3 gt200 | 0 | 0 |
| Y3 gt400 | 0 | 0 |
| Y3 Intensity | 0.16 | 0.14 |
| Y3 Coverage | 0.10 | 0.10 |

*Y1/Y3 Prevalence: Prevalence of year 1 or year 3

*Y1/Y3 Intensity: Intensity of year 1 or year 3

*Y1/Y3 gt200: Prevalence of intensity that was greater than 200 epg in year 1 or year 3

*Y1/Y3 gt400: Prevalence of intensity that was greater than 400 epg in year 1 or year 3

*Y1/Y3 Coverage: The percentage of School-aged children who received MDA in year 1 or year 3

Table S10. Variable importance of GBM and rf with Tanzania year 3 data

| Variable | Percentage of variance contribution  (GBM) | Percentage of variance contribution (rf) |
| --- | --- | --- |
| Y3 Prevalence | 0.36 | 0.22 |
| Y3 gt200 | 0.02 | 0.15 |
| Y3 gt400 | 0.04 | 0.1 |
| Y3 Intensity | 0.39 | 0.38 |
| Y3 Coverage | 0.18 | 0.15 |

*Y3 Prevalence: Prevalence of year 3

*Y3 Intensity: Intensity of year 3

*Y3 gt200: Prevalence of intensity that was greater than 200 epg in year 3

*Y3 gt400: Prevalence of intensity that was greater than 400 epg in year 3

*Y3 Coverage: The percentage of School-aged children who received MDA in year 3

Table S11. Standardized coefficients of GLMs with Tanzania year 3 data

| Variable | Coefficients  (Elastic-net logistic) | Coefficients  (LASSO) |
| --- | --- | --- |
| Y3 Prevalence | 0.33 | 0.39 |
| Y3 gt200 | 0.03 | 0 |
| Y3 gt400 | 0.18 | 0.24 |
| Y3 Intensity | 0.05 | 0 |
| Y3 Coverage | 0 | 0 |

*Y3 Prevalence: Prevalence of year 3

*Y3 Intensity: Intensity of year 3

*Y3 gt200: Prevalence of intensity that was greater than 200 epg in year 3

*Y3 gt400: Prevalence of intensity that was greater than 400 epg in year 3

*Y3 Coverage: The percentage of School-aged children who received MDA in year 3

Table S12. Variable importance of GBM and rf with Kenya and Tanzania year 1 and year 3 data (with Country label)

| Variable | Percentage of variance contribution  (GBM) | Percentage of variance contribution (rf) |
| --- | --- | --- |
| Y1 Prevalence | 0.06 | 0.06 |
| Y1 gt200 | 0.02 | 0.06 |
| Y1 gt400 | 0.02 | 0.06 |
| Y1 Intensity | 0.09 | 0.14 |
| Y1 Coverage | 0.03 | 0.04 |
| Y3 Prevalence | 0.04 | 0.07 |
| Y3 gt200 | 0.002 | 0.05 |
| Y3 gt400 | 0.12 | 0.07 |
| Y3 Intensity | 0.28 | 0.19 |
| Y3 Coverage | 0.1 | 0.14 |
| Country | 0.25 | 0.14 |

*Y1/Y3 Prevalence: Prevalence of Year 1 or Year 3

*Y1/Y3 Intensity: Intensity of Year 1 or Year 3

*Y1/Y3 gt200: Prevalence of intensity that was greater than 200 epg in Year 1 or Year 3

*Y1/Y3 gt400: Prevalence of intensity that was greater than 400 epg in Year 1 or Year 3

*Y1/Y3 Coverage: The percentage of School-aged children who received MDA in Year 1 or Year 3

Table S13. Standardized coefficients of GLMs with Kenya and Tanzania year 1 and year 3 data (with Country label)

| Variable | Coefficients  (Elastic-net logistic) | Coefficients  (LASSO) |
| --- | --- | --- |
| Country (Kenya) | -0.84 | -1.22 |
| Country (Tanzania) | 0.84 | 1.04 |
| Y1 Prevalence | -0.13 | -0.19 |
| Y1 gt200 | 0 | 0 |
| Y1 gt400 | 0.003 | 0 |
| Y1 Intensity | 0 | 0 |
| Y1 Coverage | -0.04 | -0.03 |
| Y3 Prevalence | 0.17 | 0.02 |
| Y3 gt200 | 0.30 | 0 |
| Y3 gt400 | 0.37 | 0.30 |
| Y3 Intensity | 0.48 | 1.27 |
| Y3 Coverage | -0.13 | 0 |

*Y1/Y3 Prevalence: Prevalence of year 1 or year 3

*Y1/Y3 Intensity: Intensity of year 1 or year 3

*Y1/Y3 gt200: Prevalence of intensity that was greater than 200 epg in year 1 or year 3

*Y1/Y3 gt400: Prevalence of intensity that was greater than 400 epg in year 1 or year 3

*Y1/Y3 Coverage: The percentage of School-aged children who received MDA in year 1 or year 3

Table S14. Variable importance of GBM and rf with Kenya and Tanzania year 3 data (with Country label)

| Variable | Percentage of variance contribution  (GBM) | Percentage of variance contribution (rf) |
| --- | --- | --- |
| Y3 Prevalence | 0.10 | 0.18 |
| Y3 gt200 | 0.02 | 0.21 |
| Y3 gt400 | 0.10 | 0.15 |
| Y3 Intensity | 0.49 | 0.28 |
| Y3 Coverage | 0.13 | 0.12 |
| Country | 0.17 | 0.07 |

*Y3 Prevalence: Prevalence of year 3

*Y3 Intensity: Intensity of year 3

*Y3 gt200: Prevalence of intensity that was greater than 200 epg in year 3

*Y3 gt400: Prevalence of intensity that was greater than 400 epg in year 3

*Y3 Coverage: The percentage of School-aged children who received MDA in year 3

Table S15. Standardized coefficients of GLMs with Kenya and Tanzania year 3 data (with Country label)

| Variable | Coefficients  (Elastic-net logistic) | Coefficients  (LASSO) |
| --- | --- | --- |
| Country (Kenya) | -0.47 | -0.78 |
| Country (Tanzania) | 0.47 | 0.52 |
| Y3 Prevalence | 0.11 | 0 |
| Y3 gt200 | 0 | 0 |
| Y3 gt400 | 0.49 | 0.55 |
| Y3 Intensity | 0.56 | 0.79 |
| Y3 Coverage | -0.10 | 0 |

*Y3 Prevalence: Prevalence of year 3

*Y3 Intensity: Intensity of year 3

*Y3 gt200: Prevalence of intensity that was greater than 200 epg in year 3

*Y3 gt400: Prevalence of intensity that was greater than 400 epg in year 3

*Y3 Coverage: The percentage of School-aged children who received MDA in year 3

Table S16. Variable importance of GBM and rf with Kenya and Tanzania year 1 and year 3 data

| Variable | Percentage of variance contribution  (GBM) | Percentage of variance contribution (rf) |
| --- | --- | --- |
| Y1 Prevalence | 0.09 | 0.08 |
| Y1 gt200 | 0.04 | 0.09 |
| Y1 gt400 | 0.04 | 0.06 |
| Y1 Intensity | 0.18 | 0.17 |
| Y1 Coverage | 0.02 | 0.05 |
| Y3 Prevalence | 0.04 | 0.07 |
| Y3 gt200 | 0.03 | 0.06 |
| Y3 gt400 | 0.10 | 0.11 |
| Y3 Intensity | 0.15 | 0.08 |
| Y3 Coverage | 0.32 | 0.23 |

*Y1/Y3 Prevalence: Prevalence of Year 1 or Year 3

*Y1/Y3 Intensity: Intensity of Year 1 or Year 3

*Y1/Y3 gt200: Prevalence of intensity that was greater than 200 epg in Year 1 or Year 3

*Y1/Y3 gt400: Prevalence of intensity that was greater than 400 epg in Year 1 or Year 3

*Y1/Y3 Coverage: The percentage of School-aged children who received MDA in Year 1 or Year 3

Table S17. Standardized coefficients of GLMs with Kenya and Tanzania year 1 and year 3 data

| Variable | Coefficients  (Elastic-net logistic) | Coefficients  (LASSO) |
| --- | --- | --- |
| Y1 Prevalence | -0.34 | -0.52 |
| Y1 gt200 | 0 | 0 |
| Y1 gt400 | 0.23 | 0 |
| Y1 Intensity | 0.35 | 0.77 |
| Y1 Coverage | -0.08 | -0.09 |
| Y3 Prevalence | 0.04 | 0.008 |
| Y3 gt200 | 0.20 | 0 |
| Y3 gt400 | 0.40 | 0.83 |
| Y3 Intensity | 0.32 | 0.35 |
| Y3 Coverage | -0.49 | -0.52 |

*Y1/Y3 Prevalence: Prevalence of year 1 or year 3

*Y1/Y3 Intensity: Intensity of year 1 or year 3

*Y1/Y3 gt200: Prevalence of intensity that was greater than 200 epg in year 1 or year 3

*Y1/Y3 gt400: Prevalence of intensity that was greater than 400 epg in year 1 or year 3

*Y1/Y3 Coverage: The percentage of School-aged children who received MDA in year 1 or year 3

Table S18. Variable importance of GBM and rf with Kenya and Tanzania year 3 data

| Variable | Percentage of variance contribution  (GBM) | Percentage of variance contribution (rf) |
| --- | --- | --- |
| Y3 Prevalence | 0.11 | 0.17 |
| Y3 gt200 | 0.03 | 0.12 |
| Y3 gt400 | 0.27 | 0.23 |
| Y3 Intensity | 0.31 | 0.28 |
| Y3 Coverage | 0.29 | 0.20 |

*Y3 Prevalence: Prevalence of year 3

*Y3 Intensity: Intensity of year 3

*Y3 gt200: Prevalence of intensity that was greater than 200 epg in year 3

*Y3 gt400: Prevalence of intensity that was greater than 400 epg in year 3

*Y3 Coverage: The percentage of School-aged children who received MDA in year 3

Table S19. Standardized coefficients of GLMs with Kenya and Tanzania year 3 data

| Variable | Coefficients  (Elastic-net logistic) | Coefficients  (LASSO) |
| --- | --- | --- |
| Y3 Prevalence | -0.04 | -0.04 |
| Y3 gt200 | -1.57 | -1.72 |
| Y3 gt400 | 3.27 | 3.43 |
| Y3 Intensity | 0.56 | 0.61 |
| Y3 Coverage | -0.68 | -0.69 |

*Y3 Prevalence: Prevalence of year 3

*Y3 Intensity: Intensity of year 3

*Y3 gt200: Prevalence of intensity that was greater than 200 epg in year 3

*Y3 gt400: Prevalence of intensity that was greater than 400 epg in year 3

*Y3 Coverage: The percentage of School-aged children who received MDA in year 3
